# Strategic-tuning of radiative excitons for efficient and stable fluorescent white organic light-emitting diodes

**DOI:** 10.1038/s41467-019-10104-4

**Published:** 2019-05-30

**Authors:** Zhongbin Wu, Yuan Liu, Ling Yu, Chenyang Zhao, Dezhi Yang, Xianfeng Qiao, Jiangshan Chen, Chuluo Yang, Hans Kleemann, Karl Leo, Dongge Ma

**Affiliations:** 1 Institute of Polymer Optoelectronic Materials and Devices, State Key Laboratory of Luminescent Materials and Devices, South China University of Technology, Guangzhou, 510640 People’s Republic of China; 2 Dresden Integrated Center for Applied Physics and Photonic Materials (IAPP), Technische Universität Dresden, Nöthnitzer Str. 61, Dresden, 01187 Germany; 3 Hubei Collaborative Innovation Center for Advanced Organic Chemical Materials, Hubei Key Lab on Organic and Polymeric Optoelectronic Materials, Department of Chemistry, Wuhan University, Wuhan, 430072 People’s Republic of China

**Keywords:** Electronic devices, Organic LEDs, Electronic and spintronic devices

## Abstract

The emerging thermally activated delayed fluorescence materials have great potential for efficiencies in organic light-emitting diodes by optimizing molecular structures of the emitter system. However, it is still challenging in the device structural design to achieve high efficiency and stable device operation in white organic light-emitting diodes. Here we propose a universal design strategy for thermally activated delayed fluorescence emitter-based fluorescent white organic light-emitting diodes, establishing an advanced system of “orange thermally activated delayed fluorescence emitter sensitized by blue thermally activated delayed fluorescence host” combined with an effective exciton-confined emissive layer. Compared to reference single-layer and double-layer emissive devices, the external quantum efficiency improves by 31 and 45%, respectively, and device operational stability also shows nearly fivefold increase. Additionally, a detailed optical simulation for the present structure is made, indicating the validity of the design strategy in the fluorescent white organic light-emitting diodes.

## Abstract (web summary)

Demonstrating white organic light-emitting diodes (OLEDs) based on thermally activated delayed fluorescence (TADF) emitters with high performance and operational stability remains a challenge. Here, the authors show efficient and stable white OLEDs based on a double-dopant TADF system.

## Introduction

White organic light-emitting diodes (WOLEDs) have attracted substantial attention nowadays due to their promising merits in solid-state lighting sources and full-color displays^1–3^. Enormous efforts have been devoted to efficient WOLEDs, such as employing the precise doping concentration regulation strategy^4–8^, eliminating the inserting layer between fluorescent and phosphorescent emissive regions^9^, utilizing exciplex systems^10,11^; p–i–n junctions^12^; tandem structure^13,14^; down-conversion^15^; exciton-confining structures^16^, and introducing the external heavy-atom effect^17^. According to the spin statistics, the singlet and triplet excitons can be generated by a 1:3 ratio in organic materials when the holes and electrons recombine^18,19^. As seen, the key to realize highly efficient WOLEDs is that all electrically generated excitons need to be effectively used for emission. For all phosphorescent WOLEDs, owing to the strong spin–orbit coupling, all singlet and triplet excitons can be harvested, achieving unity exciton utilization efficiency, thus the phosphorescent WOLEDs have been considered as the most promising device architecture during the past years^20^. But there is still a bottleneck to simultaneously realize high efficiency and long device lifetime in phosphorescent WOLEDs, which is restricted by the absence of the efficient and stable blue phosphorescent emitters. Then, hybrid WOLEDs combining the stable blue fluorophores and efficient long-wavelength phosphores can likewise harvest all generated excitons by the “triplet harvesting” strategy, yielding the compatibility of good stability and high efficiency^21–24^.

Recently, *E*-type delayed fluorescence^25^, i.e., thermally activated delayed fluorescence (TADF) without the metal–organic complexes has aroused researchers’ interest, which has been widely considered as a promising route to harvest nonradiative triplet excitons in OLEDs^26^. Because the energy gap between the singlet excited state and triplet excited state is sufficiently small in TADF processes, the generated nonradiative triplet excitons can be efficiently upconverted into the radiative singlet excitons by the reverse intersystem crossing (RISC) process^27–31^. TADF-based monochrome OLEDs employing pure organic aromatic compounds as emitters have realized nearly 100% exciton production efficiency and the maximum external quantum efficiency (EQE) has reached beyond 30%, and meanwhile the operational stability has improved so much^32,33^, but it is still unsatisfactory in the development of TADF-based WOLEDs. Several preliminary studies have been reported on the all-fluorescent WOLEDs. Adachi’s group^34^ pioneered TADF emitter-based fluorescent WOLEDs with a maximum EQE of 6.7% and current efficiency (CE) of 16.7 cd A^−1^. Then, they introduced a high-energy-level blue TADF molecule as the common host and conventional red/green fluorescent molecules as dopants, a maximum EQE of 12.1% was realized^35^. Zhao et al.^36^ demonstrated fluorescent WOLEDs composed of a blue TADF host and an orange fluorescent dopant with the maximum EQE of 7.48%, CE of 20.2 cd A^−1^, power efficiency (PE) of 15.9 lm W^−1^. Lee et al.^37^ doped two conventional fluorescent emitters into a TADF host to form a single-emitting layer (EML) fluorescent white device with a maximum EQE of 14.0% and a maximum PE of 36.2 lm W^−1^. Recently, Su et al. designed an efficient yellow TADF emitter combined with a blue TADF emitter (3,6-2TPA-TX). They achieved a maximum EQE of 20.4% in their fluorescent WOLED using common multi-EML structures^38^. Despite of these progress, efficient and stable TADF-based WOLEDs have not ever been achieved by adequate utilization of triplet excitons and tailored device design. Actually, it is really challenging to develop efficient and stable device design strategies for all-fluorescent WOLEDs that can render various channels to harvest all singlet and triplet excitons and achieve balanced charge injection as well as effective exciton and charge carrier confinement.

Here, we show an efficient and stable strategy using an orange TADF emitter sensitized by a high-energy-level blue TADF host. The key point is doping an orange TADF emitter with an ultralow concentration into a blue TADF emissive host, forming a double-dopant system combined with an effective exciton-confined emissive layer architecture to fully utilize all electrically generated excitons. Using this approach, extremely high-efficiency fluorescent WOLEDs with nearly 100% exciton utilization efficiency can be realized. The resulting WOLED exhibits excellent electroluminescence (EL) performance with the maximum EQE, CE, and PE of 20.5%, 51.3 cd A^−1^, and 59.6 lm W^−1^, respectively, which should be among the highest values for all-fluorescent WOLEDs. The device also shows very impressive operational stability by the proposed strategy. We also present a comprehensive optical simulation for our WOLEDs. These results indicate that proper device architecture facilitates highly effective harvesting of all generated singlet and triplet excitons in TADF-based fluorescent WOLEDs.

## Results

### Construction of the device structure

To illustrate our device design strategy, the selection of emitting dopants is a primary consideration. When the TADF-emitting dopants with a very small energy gap between *S*_1_ and *T*_1_ are used to fabricate OLEDs, the internal quantum efficiency (IQE) can be expressed as,

1 $$\eta _{{\mathrm{IQE}}} = \left[ {0.25 + \frac{{\left( {0.25\phi _{{\mathrm{ISC,D}}} + 0.75} \right)\phi _{{\mathrm{RISC}}}}}{{1 - \phi _{{\mathrm{ISC}},{\mathrm{D}}}\phi _{{\mathrm{RISC}}}}}} \right]\phi _{{\mathrm{PL,D}}}$$

where *ϕ*_ISC,D_ is the ISC efficiency, *ϕ*_RISC_ is the RISC efficiency, *ϕ*_PL,D_ is the PL quantum yield of the dopants. Therein, *ϕ*_ISC,D_ and *ϕ*_RISC_ can be calculated as follows,

2 $$\phi _{{\mathrm{ISC}},{\mathrm{D}}} = \frac{{\kappa _{{\mathrm{ISC}}}}}{{\kappa _{{\mathrm{ISC}}} + \kappa _{\mathrm{r}} + \kappa _{{\mathrm{nr}}}}}$$

3 $$\phi _{{\mathrm{RISC}}} = \frac{{\kappa _{{\mathrm{RISC}}}}}{{\kappa _{{\mathrm{RISC}}} + \kappa _{{\mathrm{nr}}}^{\mathrm{T}}}}$$

where *κ*_nr_ is the nonradiative decay rate of the singlets and *κ*_nr_^T^ is the rate for the triplet decay processes, except for RISC. As can be seen, the *ϕ*_RISC_/*ϕ*_ISC,D_ and *ϕ*_PL_ of the dopants determine IQE. Thus, TADF materials with high *ϕ*_RISC_/*ϕ*_ISC,D_ and *ϕ*_PL_ values as the emitters are necessary to realize efficient fluorescent WOLEDs. In this work, bis[4-(9,9-dimethyl-9,10-dihydroacridine)phenyl]sulfone (DMAC-DPS) for blue emission^39^ and 10-(7-fluoro-2,3-diphenylquinoxalin-6-yl)-10H-phenoxazine (FDQPXZ) for orange emission with high *ϕ*_RISC_/*ϕ*_ISC,D_ and *ϕ*_PL_ values^40^ are selected as the emitting dopants. Bis(2-(diphenylphosphino)phenyl)ether oxide (DPEPO) with a high T_1_ level of 3.30 eV acts as the host of DMAC-DPS. The well-known ambipolar material 4,4′-bis(N-carbazolyl)biphenyl (CBP) is utilized as the host for the orange emitter. To minimize the working voltage and improve the charge balance, 20 wt% hexaazatriphenylene hexacarbonitrile (HATCN) doped into 1,1′-bis[4-(di-p-tolylamino)phenyl]cyclohexane (TAPC) and 3 wt% lithium carbonate (Li_2_CO_3_) doped into 1,3-bis(3,5-dipyrid-3-yl-phenyl)benzene (BmPyPB) are used as the hole-transporting and electron-transporting layers, respectively.

### Device structure and performance

**Fig. 1:**
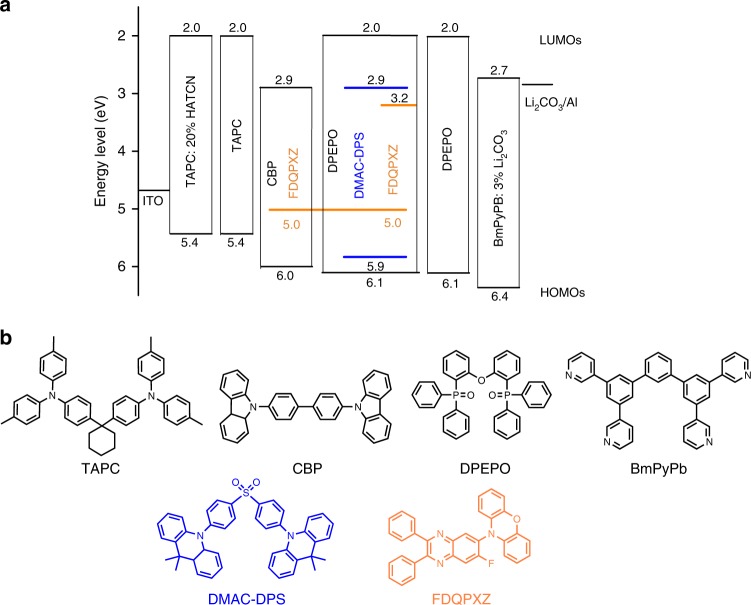
Energy-level diagram and chemical molecular structures. **a** Proposed energy-level diagram of the fluorescent white organic light-emitting diodes (WOLEDs). Note that these numbers indicate the respective highest occupied and lowest unoccupied molecular orbital (HOMO and LUMO, respectively) energies relative to the vacuum level. **b** Chemical molecular structures of the functional materials used

Figure 1 shows illustrated energy-level diagram and chemical structures of the used materials. It is noteworthy that TAPC and BmPyPB can effectively confine holes and electrons within the exciton recombination zone. On the one hand, the utilization of TAPC could prevent electrons transporting across the emissive layer, thus, substantially minimizing the electron leakage from the emissive layer because of the smaller lowest unoccupied molecular orbital (LUMO) of TAPC than that of CBP and FDQPXZ. On the other hand, we also notice that there is the energy offset (0.3 eV) of the highest unoccupied molecular orbital (HOMO) between BmPyPB and DPEPO, which will prevent the injected hole transport into the nonradiative layer, thus, effectively confines well the holes in the emissive layer. The detailed design produces an energetic well-like emissive zone. When the injected charges or generated excitons enter into the emissive region, they would not leak out or get back, increasing the likelihood that singlet and triplet excitons will radiatively decay within the emissive zone.

**Fig. 2:**
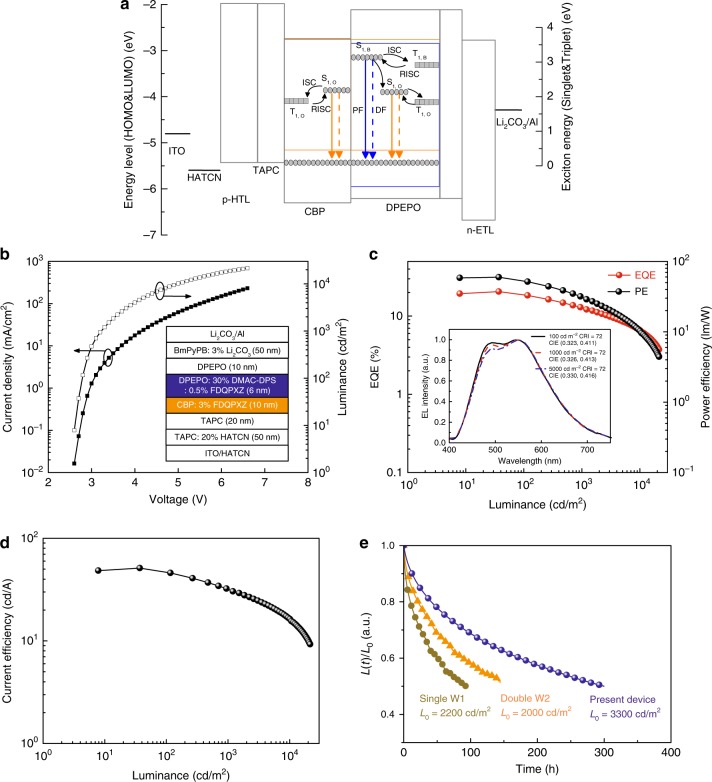
Energy transfer processes and device performance. **a** Energy transfer processes in the emissive zones of the optimized fluorescent white organic light-emitting diodes (WOLEDs). **b** Current density–voltage–luminance (*J–V–L*) characteristics of the fluorescent WOLEDs. The inset illustrates the detailed device structure of the optimized WOLEDs. **c** External quantum efficiency (EQE) and power efficiency (PE)-luminance characteristics of the fluorescent WOLED. Inset exhibits the normalized electroluminescence spectra at the different luminance of 100, 1000. and 5000 cd/m^2^, and the respective Commission Internationale de L’Eclairage (CIE) and color rendering index (CRI). **d** Current efficiency-luminance characteristics of present WOLED. **e** Device lifetime measurement of conventional single- and double-emissive layer WOLEDs, and the present WOLED with proposed emissive architecture

After optimizing the doping concentration and emissive layer thickness, our final structure is ITO/HATCN (10 nm)/TAPC: 20 wt% HATCN (50 nm)/TAPC (20 nm)/CBP: 3 wt% FDQPXZ (10 nm)/DPEPO: 30 wt% DMAC-DPS: 0.5 wt% FDQPXZ (6 nm)/DPEPO (10 nm)/BmPyPB: 3 wt% Li_2_CO_3_ (50 nm)/Li_2_CO_3_ (1 nm)/Al (100 nm). Figure 2a illustrates the proposed exciton energy-transfer processes within the emissive zone (also shown in Supplementary Fig. 1), which will be proved below. Due to the electron-transporting property of DPEPO and the superior ambipolar-transporting characteristics of CBP^41^, it can be expected that the main recombination zone should be across the interface of CBP:3% FDQPXZ and DPEPO:30% DMAC-DPS:0.5% FDQPXZ, and the broadening exciton recombination zone also benefits the improvement of efficiency roll-off. On the basis of this structural engineering via an orange TADF emitter sensitized by a high-energy-level blue TADF host, the resulting WOLED exhibits very impressive EL performances, as shown in Fig. 2b and Supplementary Fig. 2. The maximum forward-viewing EQE, CE, and PE reach 20.5%, 51.3 cd A^−1^, and 59.6 lm W^−1^, respectively. At a typical display luminance of 100 cd m^−2^, they remain as high as 18.8%, 46.5 cd A^−1^, and 52.4 lm W^−1^. All the results are obtained in the forward direction without any out-coupling techniques. Table 1 summarizes key performance parameters of the resulting WOLEDs. Figure 2c reveals a low turn-on voltage of 2.6 V and a rather low-driving voltage of 3.2 V at the brightness of 1000 cd m^−2^. In addition, the EL spectra cover all wavelengths from 400 to 780 nm (see the inset of Fig. 2b), and the CRI is calculated to reach 72. Notably, this WOLED exhibits high stability of the EL spectra. Varying the brightness from 100 to 5000 cd m^−2^ (corresponding to the driving voltage of 3–4.2 V), the WOLED shows negligible alteration of the 1931 Commission Internationale de L’Eclairage (CIE) coordinates, CIE *x* = 0.323–0.330 and CIE *y* = 0.411–0.416, thus revealing superior chromatic stability.

**Table 1:** Summary of device performances

Device	*V*_on_^a^ (V)	Max EQE^b^ [%]	Max CE^b^ [cd A^-1^]	Max PE^b^ [lm W^-1^]	CRI^c^	CIE^c^ (*x*, *y*)	Performance at the brightness of 100/1000 cd m^−2^		
							EQE [%]	CE [cd A^-1^]	PE [lm W^-1^]
Single-EML	2.7	14.1	39.4	45.8	64	(0.342, 0.453)	12.2/9.7	33.5/27.3	34.0/19.5
Double-EML	2.8	15.7	39.1	41.0	70	(0.322, 0.414)	12.3/9.3	30.4/23.0	28.4/17.3
Proposed EML	2.6	20.5	51.3	59.6	72	(0.326, 0.413)	18.8/13.0	46.5/31.9	52.4/31.7

^a^Turn-on voltages at 1.0 cd m^−2^; ^b^maximum external quantum efficiency, maximum power efficiency; ^c^Commission Internationale de L’Eclairage (CIE), color rendering index (CRI) at the brightness of 1000 cd m^−2^

**Fig. 3:**
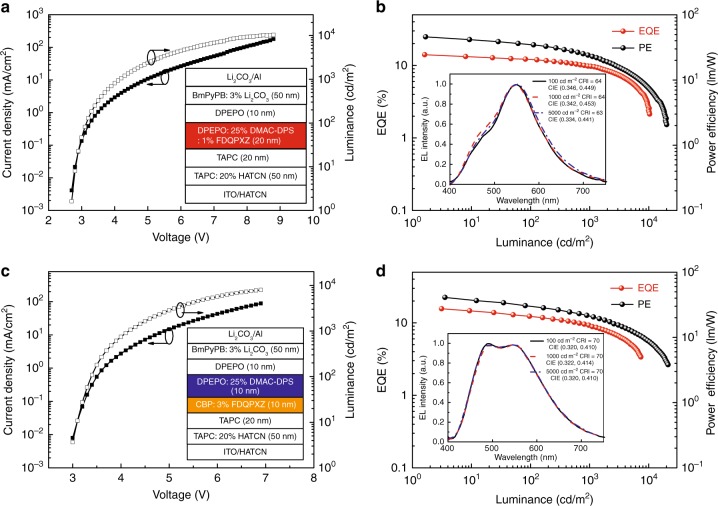
Electroluminescence performance of reference devices**. a** Current density–voltage–luminance (*J–V–L*) characteristics of the reference white organic light-emitting diodes (WOLEDs) with the single-emissive layer. The inset illustrates the detailed device structure of the optimized reference fluorescent WOLEDs. **b** External quantum efficiency (EQE) and power efficiency (PE)-luminance characteristics of the fluorescent WOLEDs. Inset exhibits the normalized electroluminescence spectra at the different luminance of 100, 1000, and 5000 cd/m^2^, and the respective Commission Internationale de L’Eclairage (CIE) and color rendering index (CRI). **c** *J–V–L* characteristics of the reference WOLED with the double-emissive layer. The inset illustrates the detailed device structure of the optimized reference fluorescent WOLEDs. **d** EQE and PE-luminance characteristics of the fluorescent WOLEDs. Inset exhibits the normalized electroluminescence spectra at different luminance, and the respective CIE and CRI

To compare with WOLEDs with conventional emissive structures, we also optimized fluorescent WOLEDs using the conventional single-emissive layer and double-emissive layer structure as the reference devices. The single-emissive layer fluorescent WOLED is ITO/HATCN (10 nm)/TAPC:20 wt% HATCN (50 nm)/TAPC (20 nm)/DPEPO:25 wt% DMAC-DPS:1 wt% FDQPXZ (20 nm)/DPEPO (10 nm)/BmPyPB:3 wt% Li_2_CO_3_ (50 nm)/Li_2_CO_3_ (1 nm)/Al (100 nm), and the conventional double-emissive layer with separated blue and orange emissive layers has the structure of ITO/HATCN (10 nm)/TAPC:20 wt% HATCN (50 nm)/TAPC (20 nm)/CBP:3 wt% FDQPXZ (10 nm)/DPEPO:25 wt% DMAC-DPS (10 nm)/DPEPO (10 nm)/BmPyPB:3 wt% Li_2_CO_3_ (50 nm)/Li_2_CO_3_ (1 nm)/Al (100 nm). Their EL performance was shown in Fig. 3 and Supplementary Fig. 2. The single-emissive layer and double-emissive layer fluorescent WOLEDs realized the maximum EQEs of 14.1% and 15.7%, CEs of 39.4 cd A^−1^ and 39.1 cd A^−1^, PEs of 45.8 lm W^−1^ and 41.0 lm W^−1^, respectively. Obviously, we can see that our proposed emissive layer with the orange TADF emitter sensitized by a blue TADF host shows much higher device performance, the maximum EQEs improve by 45 and 31%, respectively. Although the reference device with the single-emissive layer also exhibits this sensitized effect, the generated excitons in the EML can leak into the adjacent TAPC layer without the effective exciton limitation owing to the high-energy level of DPEPO, resulting in the low exciton utilization efficiency. Besides, the main exciton recombination zone in the reference device locates in a very narrow region near the interface of TAPC/EML because of the strong unipolar electron-transporting property of DPEPO, which would lead to the severe exciton quenching at high current density.

Our design strategy provides not only obvious increase of device efficiency but also a significant enhancement of operational stability in the present WOLEDs. For example, the normalized luminance of the fluorescent WOLEDs as a function of operation time at initial luminance are presented in Fig. 2e, the lifetime (T_50_), defined as the elapsed operation time at which the luminance drops to 50% of the initial value, is about 300 h for our present device at initial luminance of 3300 cd m^−2^. To predict T_50_ at *L*_0_ = 1000 cd m^−2^, an acceleration factor of 1.7 for each device from the lifetime measurements can be obtained. According to (*L*_0_)^n^ × T_50_ = constant^42,43^, T_50_ = 2283 h for our present device, 382 h for single W1 and 487 h for double W2 at the initial luminance of 1000 cd m^−2^. As seen, the lifetime of our device is almost sixfold longer than that of single W1 and nearly fivefold times longer than that of double W2, which can be attributed to more efficient exciton utilization and mitigated exciton quenching in a broadened emissive layer compared with the reference conventional devices.

Remarkably, according to our device design strategy, the performance of the optimized WOLEDs in this work significantly exceeds that of other reported all-fluorescent WOLEDs, especially for the PEs that are strongly enhanced. Supplementary Table 1 summaries the EL performances (without any out-coupling techniques) of representative fluorescent WOLEDs reported. However, like most TADF-based WOLEDs, the efficiencies still have much room that can be further improved compared with the state-of-the-art phosphorescent WOLEDs. We also notice that the orange TADF emitter in this work is not efficient enough because the photoluminescence quantum yield has not reached unity. To fully exploit the potential of our design concept, the introduction of efficient and stable ambipolar host materials for blue TADF emitters and more efficient orange/red TADF molecules with shorter excitation-state lifetime would be helpful.

### Working mechanism

**Fig. 4:**
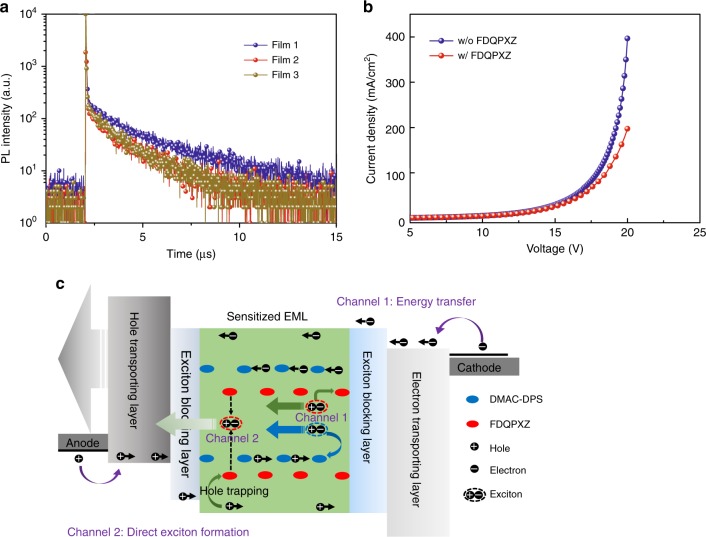
Work mechanism of the proposed devices**. a** Transient photoluminescence decay curves measured at 470 nm with an excitation wavelength of 350 nm. Film 1 consists of DPEPO:30% DMAC-DPS (20 nm); film 2 is DPEPO:30% DMAC-DPS:0.5% FDQPXZ (20 nm); film 3 is DPEPO:30% DMAC-DPS:1% FDQPXZ. **b** Current density–voltage (*J–V*) characteristics of the hole-only device with the structure of ITO/HATCN (10 nm)/TAPC:HATCN (20 wt%, 50 nm)/TAPC (20 nm)/DPEPO:25% DMAC-DPS (20 nm) or DPEPO:25% DMAC-DPS:1.0% FDQPXZ (20 nm)/TAPC (20 nm)/TAPC:HATCN (20 wt%, 50 nm)/HATCN (10 nm)/Al. **c** Illustrated schematics of the work mechanism consisting of two parallel channels: efficient energy transfer and direct exciton formation process

To study the working mechanism in the sensitized system, the transient photoluminescence (PL) behaviors and charge carrier-trapping effect were examined (see Fig. 4). We tested the transient PL of films consisting of DPEPO:30% DMAC-DPS (film 1), DPEPO:30% DMAC-DPS:0.5% FDQPXZ (film 2), DPEPO:30% DMAC-DPS:1% FDQPXZ (film 3). Clearly the film 1 illustrates obvious prompt and delayed parts in the PL decay curves, which is the classical characteristics of TADF (see Fig. 4a). When the orange TADF emitter doped into the blue-emitting layer, the lifetime and ratio of the delayed part of DMAC-DPS obviously decrease because of the efficient energy-transfer process from DMAC-DPS molecules to FDQPXZ, exhibiting the effect of orange TADF emitter sensitized by the high-energy-level blue TADF. Furthermore, to study the charge carrier-trapping effect of the orange TADF emitter in the system, we fabricated hole-only devices with the structure of ITO/HATCN (10 nm)/TAPC:20 wt% HATCN (50 nm)/TAPC (20 nm)/DPEPO:25 wt% DMAC-DPS (20 nm) or DPEPO:25 wt% DMAC-DPS:1.0 wt% FDQPXZ (20 nm)/TAPC (20 nm)/TAPC:20 wt% HATCN (50 nm)/HATCN (10 nm)/Al. When doping the orange FDQPXZ emitter into the DPEPO:25% DMAC-DPS layer, the hole current decreases obviously, indicating that FDQPXZ molecule possesses the effective hole carrier-trapping effect. This trapping effect is caused by the very large HOMO offset of 0.9 eV between the DMAC-DPS/DPEPO and FDQPXZ molecules, which will be beneficial for improving the performance because it can decrease the polaron density and thus reduce the exciton–polaron quenching especially at the low current density^7,9,12^. Thus, in the sensitized system, there are two parallel working mechanisms, one is the efficient energy transfer from the blue DMAC-DPS to orange FDQPXZ, the other is the charge carrier trapping of orange-emitter molecules. These two parallel channels cooperatively let the present WOLED utilize all of the generated singlet and triplet excitons within the exciton recombination zone.

### Optical simulation

Due to the determination of EQE parameters and the implementation of optical effects in a microcavity model, all individual light loss channels in OLEDs could be quantitatively demonstrated in detail^44,45^. Here, we provide a detailed optical simulation for our optimized WOLED to prove the nearly unitary exciton utilization efficiency. In general, to model the emissive characteristics of an OLED, it is assumed that the emissive zone contains an ensemble of mutually incoherent dipole radiators with distributions in the dipole orientations, locations, and frequencies. The electrical losses are ignored and the nonradiative losses exhibit only small variation because of the limited Purcell effect for highly efficient emitting materials^46^.

**Fig. 5:**
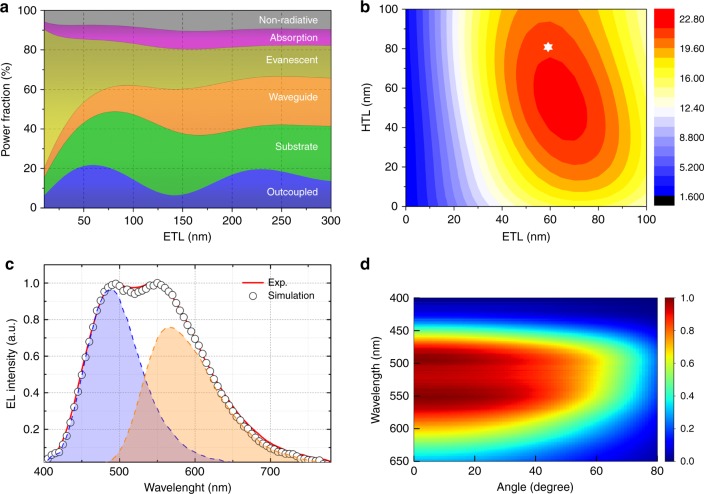
Optical simulation. **a** Simulated photon distribution of all loss channels in dependence of the electron-transporting layer (ETL) thickness in the present white organic light-emitting diodes (WOLEDs). Most generated photons remain in waveguided and evanescent modes. **b** Calculated extraction efficiency from the simulated photon distribution of the present WOLEDs, HTL denotes the hole-transporting layer. The pentagram symbol represents the theoretical maximum external quantum efficiency (21.7%) in our proposed structure. **c** The experimental and theoretical spectra of the WOLEDs according to the monochrome blue and orange spectra. **d** Angular resolved spectral radiant intensity (SRI) of the present WOLED with the electron-transporting layer thickness of 60 nm at the brightness of 1000 cd m^−2^. The displayed is the SRI in dependence of the wavelength *λ* and polar angle *θ*

The detailed simulation parameters and process are also depicted in the Supplementary Figs. 3–5 and Supplementary Note 1. The radiation characteristics of OLEDs are then obtained by the contributions over these distributions. Accordingly, Fig. 5a illustrates the relative photon distribution, i.e., the distributed quantum efficiencies of all channels in dependence of the electron-transporting layer (ETL) thickness. A theoretical maximum outcoupled EQE of 21.7% at the thickness of 60 nm can be predicted in our device structure, which is very close to the actually obtained value (20.5%). It is worth to note that around 24.0% of generated photons is restricted in the substrate mode, and these photons could be extracted by simple out-coupling techniques such as the introduction of half sphere, indicating potentially 46.0% EQE could be reached in our device structure. Figure 5b shows the calculated coupling efficiency as a function of the hole transport and electron transport layer thickness. Without considering the electrical balance in real devices, a maximum value of 22.7% can be predicted at the ETL thickness of 65 nm and hole-transporting layer (HTL) of 50 nm, which is also very close to the value of 21.7% at the ETL thickness of 60 nm in our electrically balanced device structure. Figure 5c gives the simulated spectrum at perpendicular direction with the photon contribution from blue and orange of 46 and 54%, respectively, agreeing with experiment results. Figure 5d describes the angle-distributed spectra. The spectra are also quite stable with the angle changing, promising for large angle demanding application (see Supplementary Fig. 6). From these simulated results, it is believed that nearly unity exciton utilization efficiency has been realized in our proposed emissive layer architecture.

### Exciton distribution

As seen, in the single-emissive W1 device, the blue emission intensity is low compared with the orange emission. The blue emission is effectively quenched by the orange emitter, but there is no happening in our present device. This effect should be due to following reasons: first, we broadened the exciton recombination zone, and meanwhile decreased the doping concentration of the orange emitter (FDQPXZ) and added the concentration of blue emitter (DMAC-DPS), suppressing the quenching effect. Second, we used the effective exciton-confined structure, the excitons can be effectively confined in the present device, but in a single W1 device the generated excitons in emissive layer will leak into the adjacent hole-transporting layer because of the high triplet and singlet energy level of DMAC-DPS, which has been proved by the experiment of inserting sensing materials. Third, we introduced the efficient sensitized effect, the generated nonradiative triplet excitons will be converted into the radiative singlet excitons by the efficient reverse intercrossing process of DMAC-DPS. To prove these, we studied the exciton recombination distribution in the emitting layer of our present WOLED and the reference single W1. The detailed information has been shown in the Supplementary Information (see Supplementary Figs. 7–11 and Supplementary Notes 2, 3).

## Discussion

Here, we demonstrate a universal approach to significantly improve the EL efficiencies of all-fluorescent WOLEDs based on TADF emitters. This design strategy can improve the charge balance, broaden the exciton recombination zone, and achieve the extreme utilization of singlet and triplet excitons within the EML, rendering extremely high efficiency together with good color stability, indicating their great potential for commercialization. The optical simulation shows the nearly unity exciton utilization efficiency. It is believed that our device design strategy can provide a new avenue for realizing high-performance all-fluorescent WOLEDs using TADF emitters.

## Methods

### Fabrication process of WOLEDs

Clean glass substrates pre-coated indium tin oxide (ITO) with a sheet resistance of 10 Ω per square can be commercially bought, and all devices are fabricated on these substrates. First, the ITO surface is cleaned using the detergents, and washed out with the deionized water. Then they are transferred into an oven for being dried at 120 °C for 4 h. After that, these substrates are treated with ultraviolet plasma for 20 min to decrease the ITO work function, then loaded into a deposition chamber. All functional layers are thermally evaporated grown in succession without breaking the vacuum (∼5 × 10^−6^ mbar). The detailed typical evaporated rates of the organic materials, Li_2_CO_3_, and Al are 1–2, 0.1–0.2, and 10–20 Å/s, respectively. The effective emissive area was up to 0.4 × 0.4 cm^2^, determined by the overlap between the Al and ITO electrodes.

### Electroluminescence performance characteristics of WOLEDs

Keithley source measurement unit (Keithley 2400 and Keithley 2000) combined with a calibrated silicon photodiode are used for measuring the current–voltage–brightness characteristics. The external quantum efficiency is calculated from the EL spectra, luminance and current density assuming a Lambertian distribution. All above EL performance measurements are carried out at room temperature under ambient conditions. The device lifetime measurement is performed in the glovebox filled with N_2_ at room temperature.

### Transient photoluminescence (PL) measurement

The measured films are grown on the quartz substrates. Edinburg FLS920 as the transient spectrometer and a picosecond pulsed UV-LASTER (LASTER 377) as the excitation source are used for the transient PL decay.

## Supplementary information

Supplementary Information

## Data Availability

The data that support the findings of this study are available from the corresponding author upon reasonable request.
