# Supplementary material for: Strategic-tuning of radiative excitons for efficient and stable fluorescent white organic light-emitting diodes
Source: Nat Commun. 2019 May 30;10:2380. doi: 10.1038/s41467-019-10104-4 (PMC6542840; doi:10.1038/s41467-019-10104-4)
Supplement: Supplementary file 1 — Supplementary Information [file 41467_2019_10104_MOESM1_ESM.pdf]

# Supplementary Information

## Strategic-Tuning of Radiative Excitons for Efficient and Stable Fluorescent White Organic Light-Emitting Diodes – Wu *et al.*

## Supplementary Figures

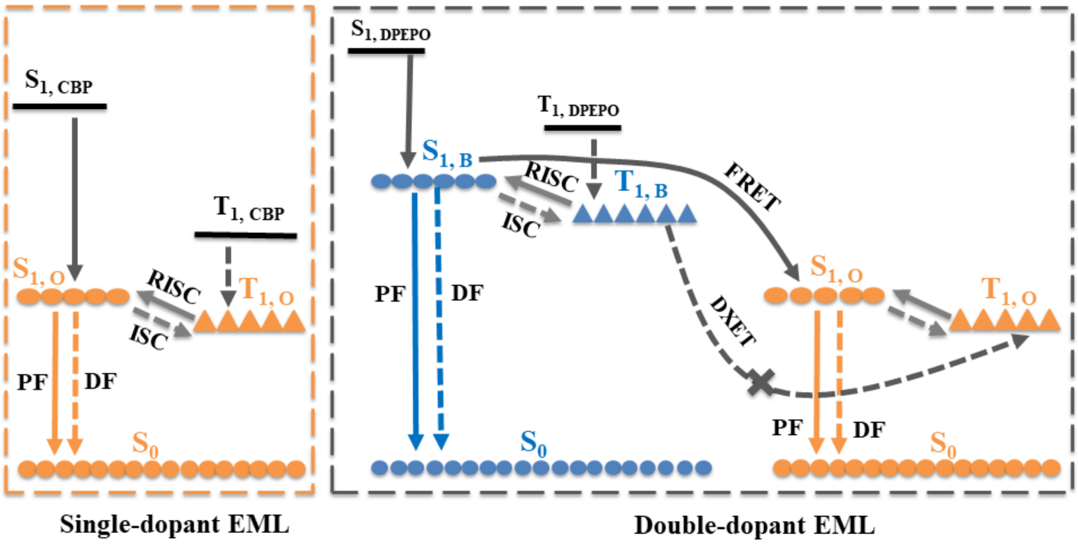


**Supplementary Figure 1 |** Schematic diagrams showing energy transfer mechanisms. FRET (Grey solid lines with the arrow) stands for Förster energy transfer, DXET (grey dash lines with the arrow) for Dexter energy transfer, PF for the prompt fluorescence, DF for the delayed fluorescence. B is the blue emitter DMAC-DPS, O is the orange emitter FDQPXZ. ISC denotes the intersystem crossing, and RISC indicates the reverse ISC.

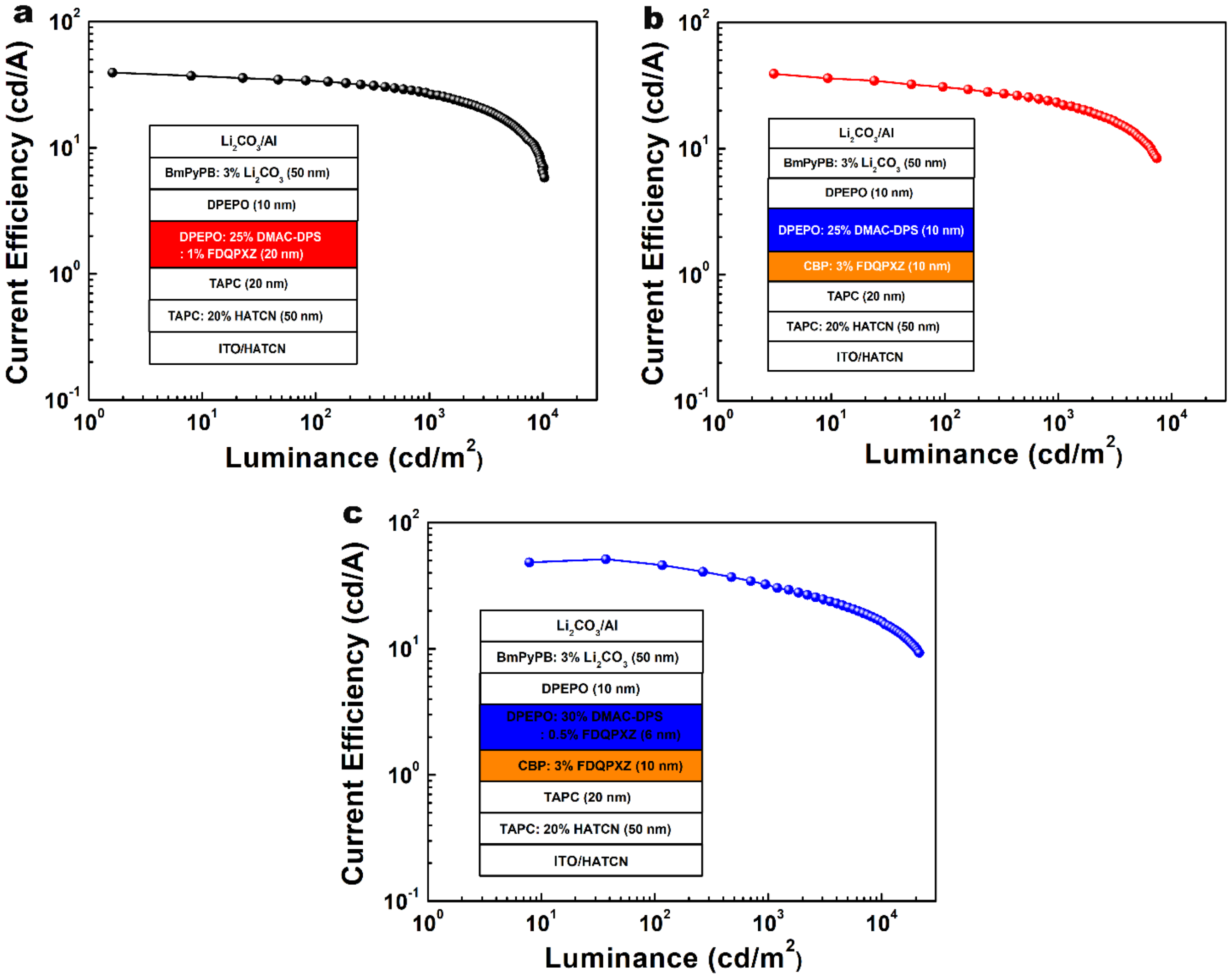


**Supplementary Figure 2 |** Current efficiency of the reference fluorescent devices. Current efficiency-luminance characteristics of the white organic light-emitting diodes (WOLEDs). a) and b) are the reference devices, single-emissive layer-based and conventional double-emitting layer-based WOLEDs, c) the proposal novel device structure using the concept of orange TADF emitter dopant sensitized by high-energy level blue thermally activated delayed fluorescence (TADF) host, realizing higher efficiency and lower efficiency roll-off. The inset exhibits the detailed device structures.

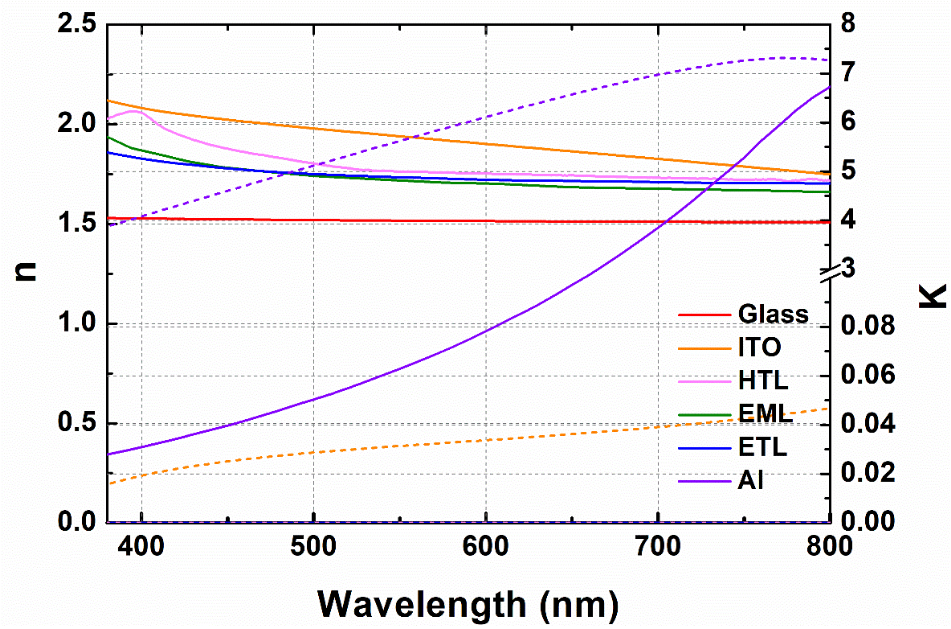


**Supplementary Figure 3 |** The refractive index of all the materials used in simulation.

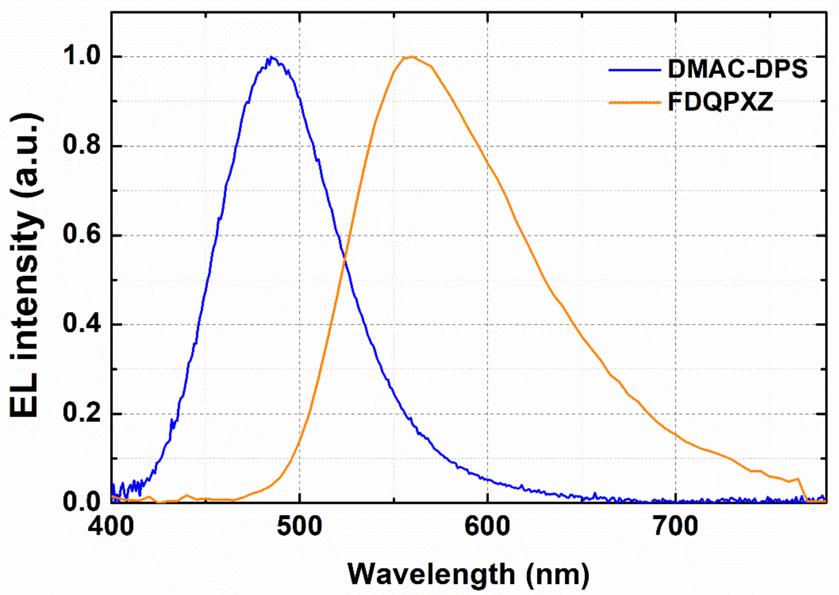


**Supplementary Figure 4 |** The emission spectra of two emitters adopted.

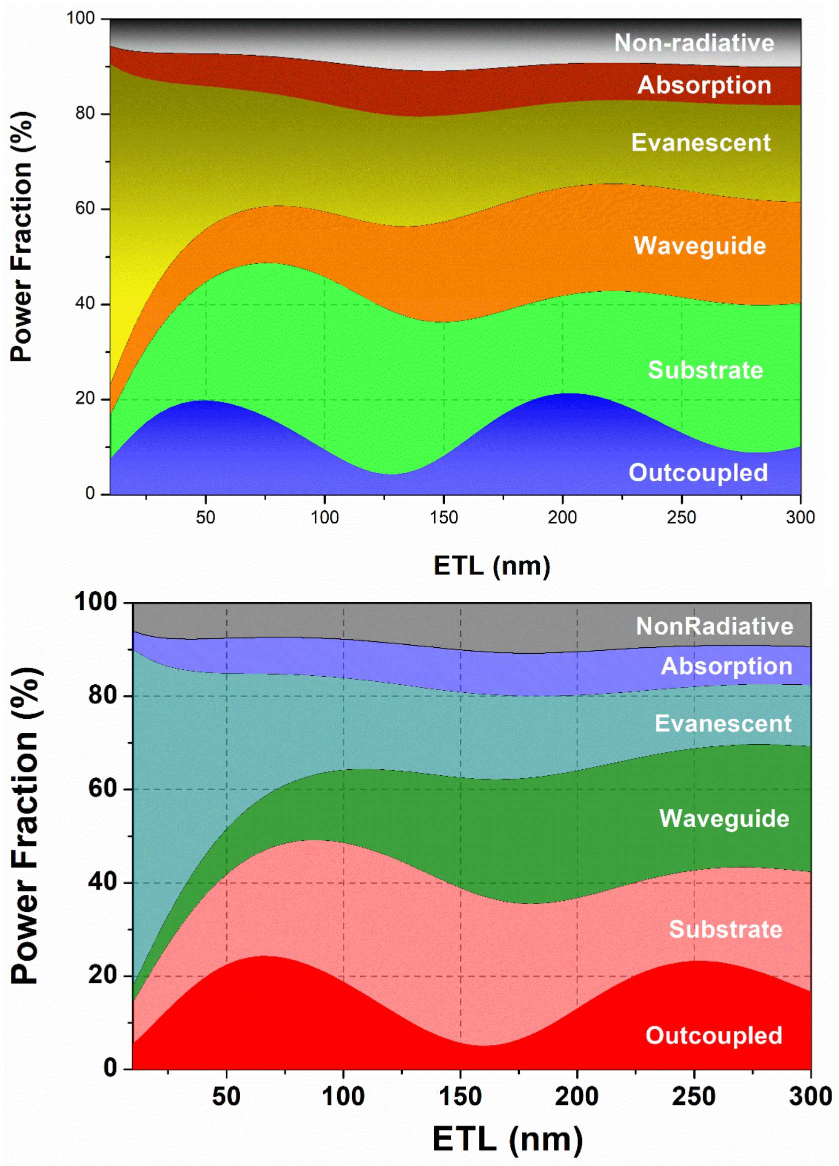


**Supplementary Figure 5 |** Simulated photon distribution. The simulated photon distribution of all loss channels in dependence of the electron-transporting layer thickness in the blue and orange organic light-emitting diodes.

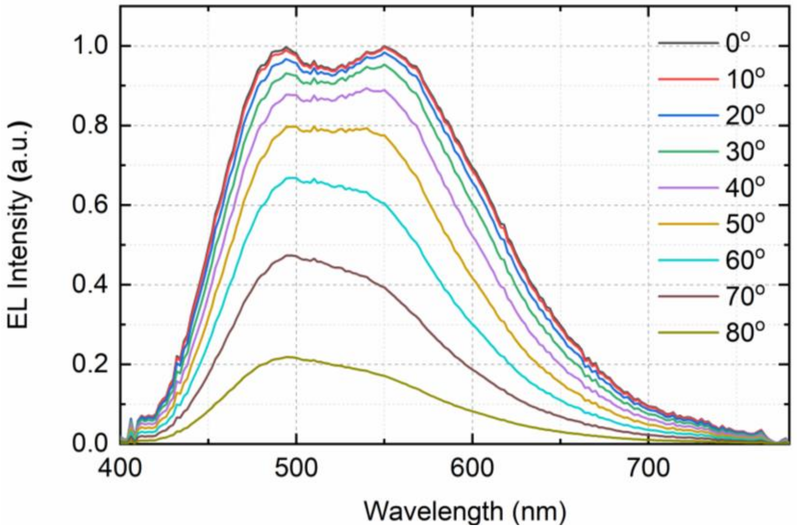


**Supplementary Figure 6 |** The flat emission spectra at different angles.

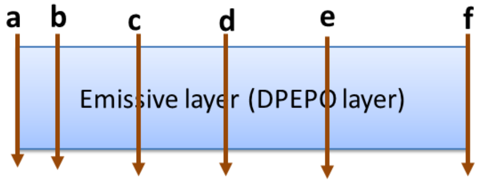


**Supplementary Figure 7 |** Position of the quenching sensing layer.

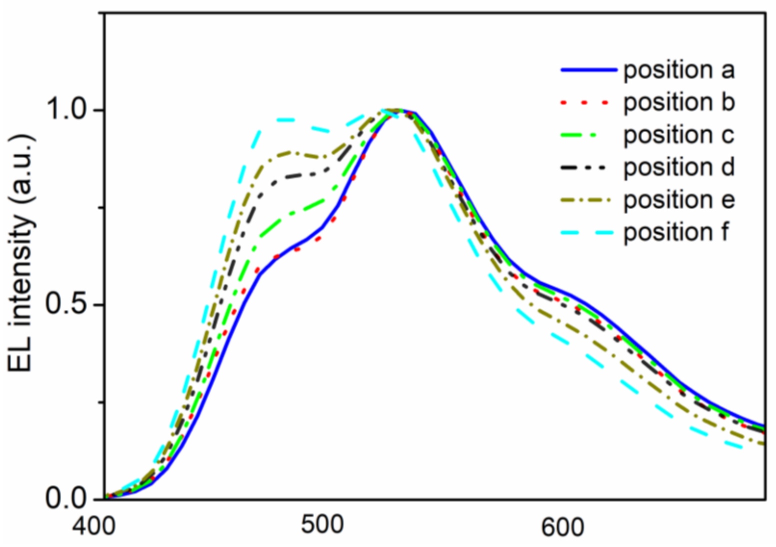


**Supplementary Figure 8 |** Normalized emission intensity after inserting the sensing layer.

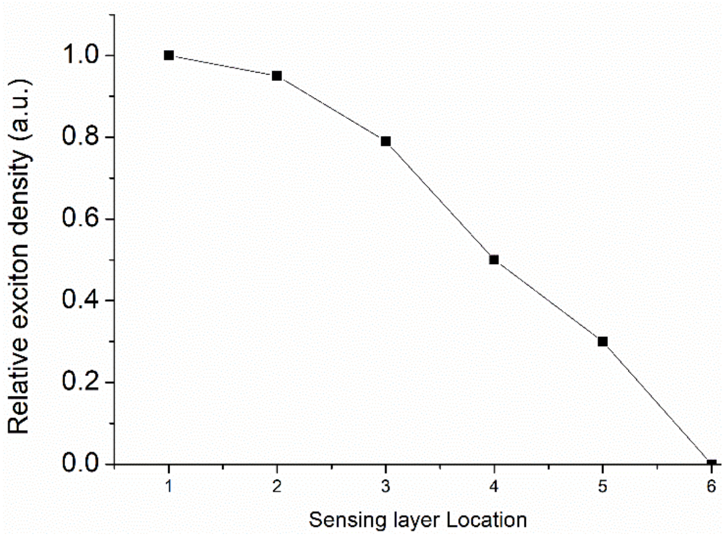


**Supplementary Figure 9 |** Relative exciton density distribution in DPEPO emitting layer.

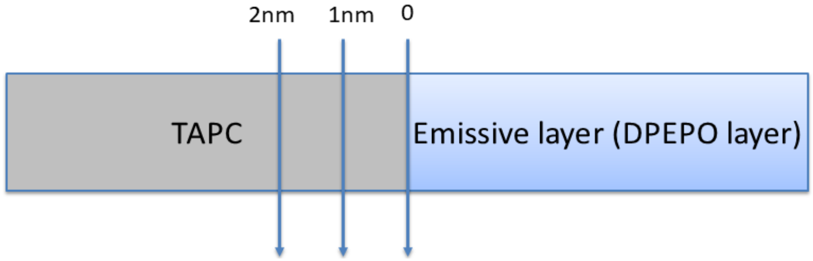


**Supplementary Figure 10 |** Position of the quenching sensing layer.

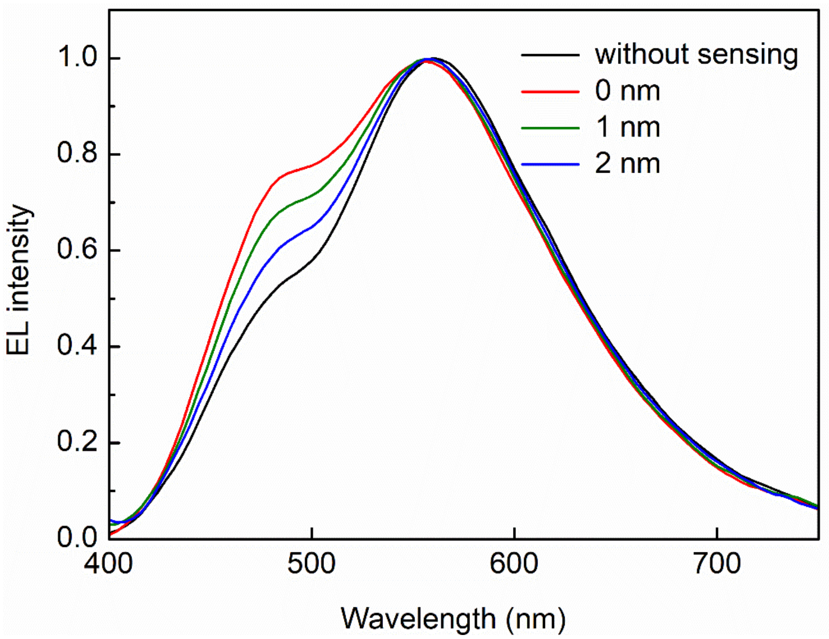


**Supplementary Figure 11 |** Emission spectra of w/ and w/o sensing layer of single W1.

## Supplementary Tables

**Supplementary Table 1 |** Summary of representative fluorescent white devices.

| WOLEDs | Ref. | $V_{on}$ [V][a)] | $EQE_{max}$ [%][b)] | $PE_{max}$ [lm/W][b)] | CIE (*x*, *y*)[c)] | CRI[c)] |
|---|---|---|---|---|---|---|
| Fluo. dopants-based | 1 | 2.8 | 18.2 | 44.6 | (0.32, 0.39) | 82 |
| | 2 | 3.0 | 12.1 | - | (0.30, 0.33) | - |
| | 3 | - | 14.0 | 36.2 | (0.31, 0.37) | - |
| | 4 | 2.5 | 7.48 | 15.9 | (0.36, 0.43) | - |
| | 5 | 4.0 | 4.7 | 6.4 | (0.34, 0.34) | - |
| | 6 | - | 15.5 | 39.3 | (0.28, 0.35) | 58.6 |
| | 7 | 3.0 | 8.4 | 15.9 | (0.41, 0.41) | 84 |
| | 8 | 2.8 | 14.0 | 48.0 | (0.37, 0.48) | 52 |
| TADF emitters-based | This work | 2.5 | 20.5 | 59.6 | (0.33, 0.41) | 72 |
| | 9 | 5.0 | 6.7 | - | (0.32,0.39 ) | - |
| | 10 | 3.6 | 17.1 | 33.4 | (0.30, 0.38) | - |
| | 11 | 3.9 | 19.1 | 40.6 | (0.32,0.43 ) | - |
| | 12 | 3.4 | 19.2 | 47.5 | (0.35, 0.46) | 69 |
| | 12 | 3.4 | 15.6 | 28.9 | (0.36, 0.38) | 95 |
| | 13 | 3.5 | 12.5 | 27.1 | (0.38, 0.40) | 71 |
| Exciplex-based | 15 | 4.0 | 11.6 | 15.8 | (0.29, 0.35) | 70.6 |
| | 16 | - | 6.2 | 10.4 | (0.45, 0.39) | - |
| | 16 | - | 7.1 | 11.4 | (0.31, 0.41) | - |

[a)]Turn-on voltages at 1.0 cd $m^{-2}$; [b)]Maximum external quantum efficiency, maximum power efficiency; [c)]Commission Internationale de L'Eclairage, color rendering index.

## Supplementary Notes

**Supplementary Note 1**: The optical simulation.

For simulation process, we adopted a classical electromagnetic model by treating the radiative dipole as an electrical dipole antenna and utilizing the transfer matrix method. The detail simulation process could be found in PHYSICAL REVIEW B 85, 115205 (2012). All the layers used here are isotropic and the emitting layer does not absorb any light. For simplification, the device structure used for simulation is Glass/ITO/HTL (Vary)/EML (10 nm)/recombination zone (0 nm)/EML (6 nm)/ETL (Vary)/Al (100 nm). The refractive index of all the materials used in the simulation is shown in Supplementary Figure 3 and the emission spectra of the two emitters adopted displayed in Supplementary Figure 4. The power fractions of all channels of the blue and orange emitters-based OLED are also shown in Supplementary Figure 5.

**Supplementary Note 2**: The exciton distribution.

To study the exciton distribution in the DPEPO emissive layer, here we use an ultrathin quenching sensing layer (0.1 nm) to ensure that the inclusion of the sensing strip does not vastly influence the charge transport. iridium bis(4-phenylthieno-[3,2-c]pyridinato-N,C20)acetylacetonate (PO-01) was chosen as the sensing layer because it has very low triplet energy level of 2.2 eV, and the relative emission intensity can provide some information about the exciton spatial distribution.

We insert 0.1 nm-thickness PO-01 into the different positions in the emissive layer, the location of the sensing layer is shown in Supplementary Figure 7. Position f represents the interfaces of EML and electron transporting layer, and position a is the interface of CBP and DPEPO emissive layers, the distance among the positions are 1 nm (ab), 1 nm (bc), 1 nm (cd), 1 nm (de), and 2 nm (ef), respectively. The EL spectra of these devices are depicted in Fig. S8, it can be seen that the blue emission at positions a and b decreases much due to the insertion of the sensing quenching layer, position f shows the nearly consistent spectrum compared to the device without the sensing layer. We also estimated the relative exciton distribution in this emissive layer, as shown in Supplementary Figure 9. Numbers 1-6 represent the positions of a-f. Here we normalized the max. exciton intensity at position a. The exciton distribution is mainly close to the interface of CBP and DPEPO layer, which means all generated excitons in our present device can be effectively confined into the emissive layer and provides the possibility of nearly 100% exciton utilization efficiency.

**Supplementary Note 3**: Examining the exciton leakage in single emissive devices.

To prove the formed excitons leaking into the adjacent TAPC layer, here we added the sensing experiment. We introduced the sensing emitting material (0.1 nm DMAC-DPS) into TAPC, the change of EL spectra will give us information about the exciton distribution. Here we insert the sensing layer into TAPC, 0, 1, 2 nm represent the distance between the sensing layer and TAPC/DPEPO-EML interface, shown as Supplementary Figure 10.

The EL spectra of these devices are depicted in Supplementary Figure 11. We can see that at the interface of DPEPO/TAPC, the EL intensity increased much compared to the device without inserting sensing layer, this is because of the electron-transporting property of DPEPO, the exciton mainly formed at this interface. When inserting the sensing layer at the 1 nm and 2 nm away this interface, the intensity then decreased but it is still higher than that of device without sensing layer, due to the exciton leaking into TAPC layer. Thus, from this sensing experiment, it can be seen that the formed excitons in the single W1 can leak into the adjacent TAPC layer, leading to low exciton utilization efficiency.

## Supplementary References


1. Wu, Z. et al. Managing excitons and charges for high-performance fluorescent white organic light-emitting diodes. *ACS Appl. Mater. Interfaces* **8**, 28780-28788 (2016).
2. Higuchi, T., Nakanotani, H. & Adachi, C. High-efficiency white organic light-emitting diodes based on a blue thermally activated delayed fluorescent emitter combined with green and red fluorescent emitters. *Adv. Mater.* **27**, 2019-2023 (2015).
3. Song, W., Lee, I. & Lee, J. Y. Host engineering for high quantum efficiency blue and white fluorescent organic light-emitting diodes. *Adv. Mater.* **27**, 4358-4363 (2015).
4. Zhao, B. et al. Highly efficient and color stable single-emitting-layer fluorescent WOLEDs with delayed fluorescent host. *Org. Electron.* **23**, 208-212 (2015).
5. Meng, L., Wang, H., Wei, X., Lv, X., Wang, Y. & Wang, P. White organic light emitting diodes based on a yellow thermally activated delayed fluorescent emitter and blue fluorescent emitter. *RSC Adv.* **5**, 59137-59141 (2015).
6. Song, W., Lee, I., Hwang, S. & Lee, J. Y. High efficiency fluorescent white organic light-emitting diodes having a yellow fluorescent emitter sensitized by a blue thermally activated delayed fluorescent emitter. *Org. Electron.* **23**, 138-143 (2015).
7. Zhang, Z. et al. High efficiency fluorescent white organic light-emitting diodes having a yellow fluorescent emitter sensitized by a blue thermally activated delayed fluorescent emitter. *Org. Electron.* **10**, 491-495 (2009).
8. Wu, Z. et al. Management of singlet and triplet excitons: a universal approach to high-efficiency all fluorescent WOLEDs with reduced efficiency roll-off using a conventional fluorescent emitter. *Adv. Opt. Mater.* **4**, 1067-1074 (2016).
9. Lee, S. Y., Yasuda, T., Yang, Y. S., Zhang, Q. & Adachi, C. Luminous butterflies: efficient exciton harvesting by benzophenone derivatives for full-color delayed fluorescence OLEDs. *Angew. Chem. Int. Ed.* **53**, 6402-6406 (2014).
10. Nishide, J., Nakanotani, H., Hiraga, Y. & Adachi, C. High-efficiency white organic light-emitting diodes using thermally activated delayed fluorescence. *Appl. Phys. Lett.* **104**, 233304 (2014).
11. Li, J. et al. A significantly twisted spirocyclic phosphine oxide as a universal host for high-efficiency full-color thermally activated delayed fluorescence diodes. *Adv. Mater.* **28**, 3122-3130 (2016).

12. Li, X. et al. High-efficiency WOLEDs with high color-rendering index based on a chromaticity-adjustable yellow thermally activated delayed fluorescence emitter. *Adv. Mater.* **28**, 4614-4619 (2016).
13. Chen, Y. et al. Highly efficient white light-emitting diodes with a bi-component emitting layer based on blue and yellow thermally activated delayed fluorescence emitters. *J. Mater. Chem. C* **6**, 2951-2956 (2018).
14. Li, Y. et al. Design strategy of blue and yellow thermally activated delayed fluorescence emitters and their all-fluorescence white OLEDs with external quantum efficiency beyond 20%. *Adv. Funct. Mater.* **26**, 6904-6912 (2016).
15. Hung, W.-Y. et al. The first tandem, all-exciplex-based WOLED. *Sci. Rep.* **4**, 5161 (2014).
16. Zhang, T. et al. Simple structured hybrid WOLEDs based on incomplete energy transfer mechanism: from blue exciplex to orange dopant. *Sci. Rep.* **5**, 10234 (2015).
